# OX_1_ and OX_2_ orexin/hypocretin receptor pharmacogenetics

**DOI:** 10.3389/fnins.2014.00057

**Published:** 2014-05-06

**Authors:** Miles D. Thompson, Henri Xhaard, Takeshi Sakurai, Innocenzo Rainero, Jyrki P. Kukkonen

**Affiliations:** ^1^University of Toronto Epilepsy Research Program, Department of Pharmacology, University of TorontoToronto, ON, Canada; ^2^Faculty of Pharmacy, Centre for Drug Research, University of HelsinkiHelsinki, Finland; ^3^Department of Molecular Neuroscience and Integrative Physiology, Faculty of Medicine, Kanazawa UniversityKanazawa, Japan; ^4^Department of Neuroscience, University of TurinTorino, Italy; ^5^Biochemistry and Cell Biology, Department of Veterinary Biosciences, University of HelsinkiHelsinki, Finland

**Keywords:** orexin, hypocretin, G protein-coupled receptor, polymorphism, pharmacogenetics

## Abstract

Orexin/hypocretin peptide mutations are rare in humans. Even though human narcolepsy is associated with orexin deficiency, this is only extremely rarely due to mutations in the gene coding prepro-orexin, the precursor for both orexin peptides. In contrast, coding and non-coding variants of the OX_1_ and OX_2_ orexin receptors have been identified in many human populations; sometimes, these have been associated with disease phenotype, although most confer a relatively low risk. In most cases, these studies have been based on a candidate gene hypothesis that predicts the involvement of orexins in the relevant pathophysiological processes. In the current review, the known human OX_1_/*HCRTR1* and OX_2_/*HCRTR2* genetic variants/polymorphisms as well as studies concerning their involvement in disorders such as narcolepsy, excessive daytime sleepiness, cluster headache, polydipsia-hyponatremia in schizophrenia, and affective disorders are discussed. In most cases, the functional cellular or pharmacological correlates of orexin variants have not been investigated—with the exception of the possible impact of an amino acid 10 Pro/Ser variant of OX_2_ on orexin potency—leaving conclusions on the nature of the receptor variant effects speculative. Nevertheless, we present perspectives that could shape the basis for further studies. The pharmacology and other properties of the orexin receptor variants are discussed in the context of GPCR signaling. Since orexinergic therapeutics are emerging, the impact of receptor variants on the affinity or potency of ligands deserves consideration. This perspective (pharmacogenetics) is also discussed in the review.

## Introduction

The orexin/hypocretin system was identified by two groups. de Lecea with colleagues described two putative peptide transmitters, encoded by a propeptide, the gene for which is located at human chromosome 17q21.2 (de Lecea et al., 1998). The hypocretin peptides were named for their expression in the synaptic vesicles in the hypothalamus and for the homology of hypocretin-2/orexin-B with some incretin peptides. The hypocretin-2/orexin-B peptide was shown to be markedly neuroexcitatory in neuronal cultures (de Lecea et al., 1998). Contemporaneously, Sakurai and co-workers deorphanized the putative G protein-coupled receptor (GPCR), HFGAN72, and identified the two peptide transmitters that activated the receptor, the common precursor peptide and its gene and, finally, a second receptor based on a sequence homology search (Sakurai et al., 1998). Peptide–receptor pharmacology was established and mRNA expression and peptide distribution in the central nervous system (CNS) was mapped.

Orexin-A/hypocretin-1 and orexin-B/hypocretin-2 are 33- and 28-residue hypothalamic peptides, respectively, derived from a 130 (or 131, depending on species)-amino acid precursor, prepro-orexin (PPO). The peptides were found to be linked to the regulation of feeding behavior based on evidence that they stimulated food intake upon intracerebroventricular administration, and increased peptide mRNA expression in the hypothalamus upon fasting. Sakurai et al. termed the peptides orexins for their orexinergic function, and the receptors OX_1_ and OX_2_ receptors (Sakurai et al., 1998).

Subsequently, genetic and disease-based studies supplied major findings concerning the physiological role of orexins in the regulation of wakefulness and sleep pattern. Mignot and co-workers isolated two OX_2_ receptor gene frame-shift mutations responsible for hereditary canine narcolepsy (Lin et al., 1999). The frame shifts generate a premature in-frame stop codon, and the truncated receptors remain cytosolic and do not traffic at all to the plasma membrane (Hungs et al., 2001). Simultaneously, Yanagisawa and co-workers showed that knockout of the precursor peptide, PPO, causes narcoleptic phenotype in mice (Chemelli et al., 1999). In 2000, a report found orexin-A to be at very low or undetectable levels in the cerebrospinal fluid (CSF) of human narcoleptics with cataplexy (Nishino et al., 2000). In contrast, mutations in PPO gene have been identified in only a few patients (Peyron et al., 2000; Gencik et al., 2001).

Further studies have established that the neurobiology of the orexin system is complex, having possible roles in stress responses, reward/addiction, analgesia, in addition to sleep and wakefulness and appetite/metabolism (Kukkonen, 2013). In the CNS, the complexity is primarily created by the wide range of orexin neuron projections. On the cellular level, orexin receptor activation produces highly diverse cellular signals. This is likely a result of orexin receptor coupling to several families of heterotrimeric G proteins and other proteins that ultimately regulate entities such as ion channels, phospholipases and protein kinases, which impact on neuronal excitation, synaptic plasticity, and cell death, to mention a few (Kukkonen, 2013; Kukkonen and Leonard, 2014; Leonard and Kukkonen, 2014). The selection of signal cascade in each case is likely determined by the expression profile of signaling components, signal complexes and other concurrent signals. The possibly distinct role of the two orexin peptides and two orexin receptors is not resolved.

**Figure 1:**
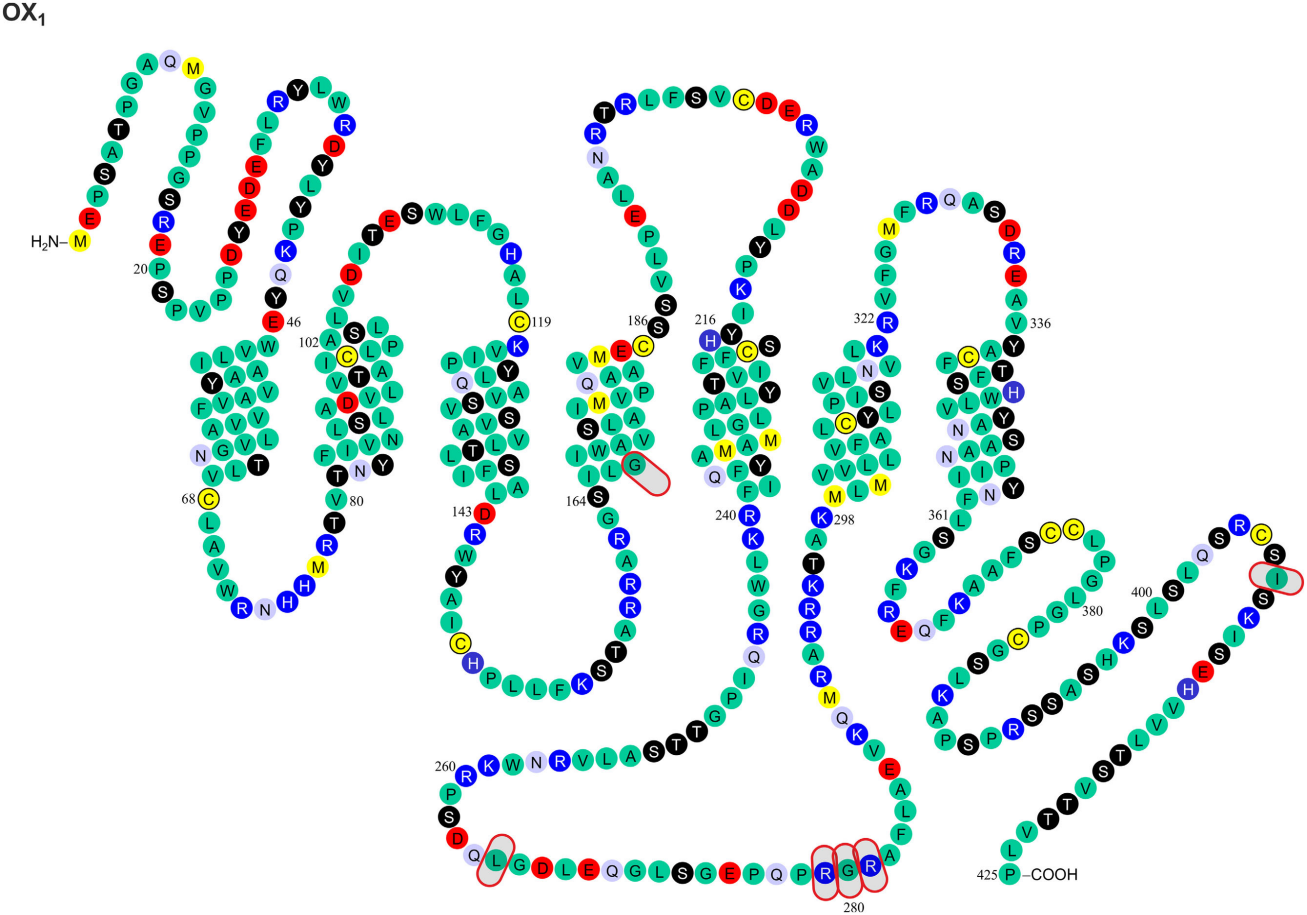
**Known amino acid sequence-changing variants of human OX_1_ receptor**. The variants are OX_1_^167^ Gly/Ser, OX_1_^265^ Leu/Met, OX_1_^279^ Arg/Glu, OX_1_^280^ Gly/Ala, OX_1_^281^ Arg/His, OX_1_^408^ Ile/Val, OX_2_^10^ Pro/Ser, OX_2_^11^ Pro/Thr, OX_2_^193^ Cys/Ser, OX_2_^293^ Ile/Val, OX_2_^308^ Val/Ile, and OX_2_^401^ Thr/Ile. The findings originate from Peyron et al. (2000) and Olafsdottir et al. (2001). The figure is modified from Kukkonen, J. P. (2013). Physiology of the orexinergic/hypocretinergic system: a revisit in 2012. *Am. J. Physiol. Cell. Physiol*. 301, C2–C32, © 2013. The American Physiological Society (Kukkonen, 2013).

**Figure 2:**
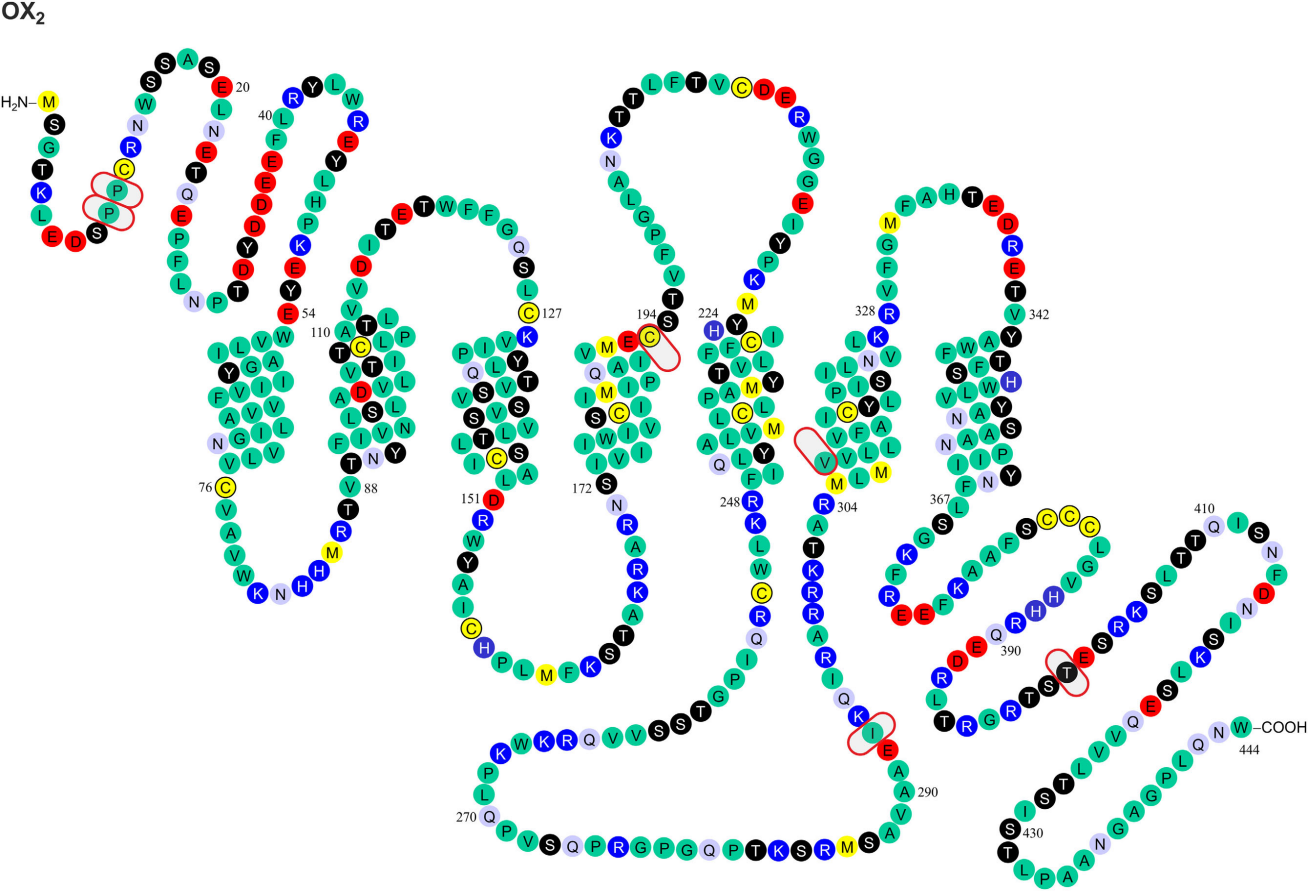
**Known amino acid sequence-changing variants of human OX_2_ receptor**. The variants are OX_2_^10^ Pro/Ser, OX_2_^11^ Pro/Thr, OX_2_^193^ Cys/Ser, OX_2_^293^ Ile/Val, OX_2_^308^ Val/Ile, and OX_2_^401^ Thr/Ile. The findings originate from Peyron et al. (2000) and Olafsdottir et al. (2001). The figure is modified from Kukkonen, J. P. (2013). Physiology of the orexinergic/hypocretinergic system: a revisit in 2012. *Am. J. Physiol. Cell. Physiol*. 301, C2–C32, © 2013. The American Physiological Society (Kukkonen, 2013).

Gene variants for the orexin peptides and, especially, their G protein-coupled receptors, OX_1_ and OX_2_, have been identified (Figures 1, 2), and investigated in many CNS disorders, including sleep and wakefulness (Lin et al., 1999), polydipsia in schizophrenia (Meerabux et al., 2005; Fukunaka et al., 2007), panic disorder (Annerbrink et al., 2011), mood disorders (Rainero et al., 2011a), migraine (Schürks et al., 2007b; Rainero et al., 2011b), and cluster headache (Rainero et al., 2004). The associations under investigation will ultimately benefit from the clarification, possible from genome-wide association studies (GWAS).

In the current report, we review the known gene variants of orexin receptors. The major focus is on the investigations of association of the variants with disease phenotypes. Before presenting those data, we present the theoretical basis of the impact of DNA and amino acid sequence variations on the protein (here orexin receptor) expression, structure, function and regulation. In general, however, definitive evidence for the functional significance of orexin receptor variants is lacking.

## How can genetic variation affect peptide transmitters and their receptors?

Gene sequence variation for a peptide transmitter may cause alterations in signaling at several stages. The first level could be the gene structural level, i.e., sequence effect on the chromatin structure with impact on the transcriptional activity. mRNA stability and processing is affected by its secondary structure. Translation may proceed at different pace depending on the codon usage. For correct translation, packing and proteolytic processing, the signal sequence is of great importance. Finally, the sequence may affect the structure and the breakdown process of the transmitter. For receptors, although mRNA issues are similar, there are specific alterations in the potentially very vulnerable processes of protein processing (folding, glycosylation), trafficking (including wrong subcellular localization), dimerization, ligand binding, signaling, and desensitization and down-regulation. A significant bottle-neck of the production lies in the folding and processing; some other issues, such as codon usage, may be of minor importance.

Since there is limited knowledge of the processing of orexin peptides and receptors, and few studies that evaluate orexin receptor variants in an expression system, it is difficult to predict a role for most of these processes for the orexin peptides or receptors. One notable exception is the Leu16Arg mutant of the signal peptide of human PPO, which impairs its processing (Peyron et al., 2000). The best characterized orexin receptor mutants are the three OX_2_ mutants found in narcoleptic canines: the two most extreme ones cause truncation and subsequent gross protein folding failure, while the Glu54Lys variant is associated with proper membrane localization but shows a notable loss of ligand binding and dramatically diminished calcium signaling (Hungs et al., 2001). While these mutations (recessively) produce a strong narcoleptic phenotype in affected animals, the human receptor variants seem to confer significantly milder phenotypes (see below).

### Impact of the genetic variation on the orexin receptor ligand binding

The most dramatic GPCR mutations—if we disregard the grossly disruptive mutations such as truncations—may result in (a) altered sensitivity to native ligands or drugs (e.g., variants of follicle-stimulating hormone receptor causing ovarian dysgenesis; Thompson et al., 2008b) or (b) disruption to the receptor's interaction with the signal transduction machinery (e.g., G protein-binding site mutations found in the β^1^-adrenoceptor; Thompson et al., 2008c). GPCRs share a common three dimensional structure composed of seven transmembrane helices (TMs). Like other family A GPCRs, orexin receptors are assumed to bind their peptide and non-peptide ligands mainly in a partially hydrophilic, partially hydrophobic cleft with possible contribution from more extracellular portion of the receptor. While orexin receptor crystal structures have not been published, mutagenesis studies have been performed and receptor models constructed (Malherbe et al., 2010; Putula et al., 2011; Tran et al., 2011; Heifetz et al., 2012; Putula and Kukkonen, 2012). Out of the 11 variable sites found and described in this review, only three—OX_1_^167^ [transmembrane helix (TM) 4, closer to intracellular side of receptor], OX_2_^193^ (TM) 4, near the putative binding cavity and OX_2_^308^ (TM6, closer to intracellular side) (Figures 1, 2)—are within the predicted transmembrane helix domain forming this cavity. Among these sites, only OX_2_^193^ is located in the putative binding cleft, where it might have a direct effect on receptor pharmacology. The other two sites are less likely to act through direct molecular contacts since they are located toward the intracellular ends of TM regions. Unfortunately, the impact of these polymorphisms has not been determined in heterologous expression studies, and neither have these sites been targeted in the point-mutation studies (Malherbe et al., 2010; Tran et al., 2011; Heifetz et al., 2012).

Most GPCR polymorphisms are found in the loops and in the N- and C-terminal. This is consistent with general observation that loops connecting helices are much more variable—and in some case hypervariable—in comparison to the transmembrane core of GPCRs (Madsen et al., 2002; Jaakola et al., 2005), and does not as such necessarily imply a functional significance. However, amino acids located outside the (predicted) binding cavity may also have consequences on the binding affinities measured in pharmacological assays, as exemplified by the canine OX_2_ Glu54Lys mutation (see above) (Hungs et al., 2001). Polymorphism at the N-terminal of OX_2_ (Pro10Ser and Pro11Thr; Figures 1, 2) may, similarly, act directly on ligand binding or indirectly via receptor structure. However, also other effects are possible, as discussed below.

Orexin-B was 2.7-fold less potent as an activator of the OX_2_^10^ Ser receptor by comparison with the wild-type OX_2_^10^ Pro receptor in calcium measurements in recombinant COS-7 cells (Thompson et al., 2004). However, this may also relate to the signaling or receptor expression/maturation; as a result, no firm conclusions can be drawn as yet, although the finding is interesting.

### Impact of the genetic variation on orexin receptor signaling

Orexin receptors are able to couple to multiple G proteins. Experimental evidence suggests that OX_2_ receptors in human adrenal cortex activate G_i_, G_s_, and G_q_ proteins (Karteris et al., 2001; Randeva et al., 2001). Mixed orexin receptor populations in rat adrenal cortex or hypothalamus couple to G_i_, G_o_, G_s_, and G_q_ (Karteris et al., 2005). In recombinant systems, OX_1_ receptors easily couple to all these three G protein families (Holmqvist et al., 2005; Magga et al., 2006). In summary, both orexin receptors are likely capable of coupling to G_i/o_, G_s_, and G_q_ family G proteins; however, this may be subject to tissue- and context-specific regulation (Kukkonen, 2013). For G protein coupling, the most central receptor domains are usually the 2nd and 3rd intracellular loops, while also the 1st intracellular loop and the receptor's C-terminus are sometimes implicated based on mutagenesis studies (Wess, 1998); orexin receptors themselves have not been examined for this. Like other GPCRs (Ritter and Hall, 2009), orexin receptors may also couple to other proteins, like β-arrestin and dynein light chain Tctex-type 1 (Milasta et al., 2005; Dalrymple et al., 2011; Duguay et al., 2011). Coupling to both these proteins is suggested to take place on the receptor's C-terminus. Variations in these regions may directly impact orexin receptor interaction with effectors while also indirectly modifying effector coupling as a result of alterations in the receptor configuration that determines the specificity of these interactions. Among the identified variable sites, OX_1_^265^, OX_1_^279^, OX_1_^280^, OX_1_^281^, OX_1_^408^, OX_2_^293^, and OX_2_^401^ (Figures 1, 2) may thus be implicated in G protein and/or other protein coupling of orexin receptors.

Further complexity in the trafficking, ligand interaction and signaling of GPCR is introduced by the fact that many GPCRs have been shown to dimerize (Bulenger et al., 2005; Milligan, 2009). In fact, some models predict that all functional GPCRs form dimers. Orexin receptors are known to homo- and hetero-dimerize/oligomerize in recombinant expression systems (Ellis et al., 2006; Ward et al., 2011; Xu et al., 2011; Jäntti et al., 2014). It should be noted, however, that there is no evidence for such interactions in native cells thus far.

GPCRs may utilize both extracellular and intracellular parts as well as the hydrophobic outer surfaces of the transmembrane helices for interaction during dimerization (see, e.g., Liang et al., 2003; Wu et al., 2010; Huang et al., 2013). One region involved in receptor–receptor interaction, at least according to X-ray crystal structure-based modeling of the CXCR4 (Wu et al., 2010) and the β_1_-adrenoceptor (Huang et al., 2013), is near the palmitoylated C-terminal region. An analogous structure is present in the human orexin receptors. It may form a coiled coil motif in the putative helix 8 (parallel to membrane) that would allow OX_1_ and OX_2_ dimerization. The impact of OX_1_^408^ and OX_2_^401^ (Figures 1, 2) on receptor dimerization is unknown; in any case, they are well downstream from the potential palmitoylation sites. The importance of dimerization/oligomerization for most GPCRs, however, is unclear (Bulenger et al., 2005; Milligan, 2009). Notable exceptions to this among GPCR family A receptors are the opioid receptors, whose pharmacology and trafficking is significantly affected by dimerization. OX_1_–CB_1_ dimerization was suggested to strongly potentiate orexin receptor signaling, but a likely explanation for the signal potentiation is, instead, offered by the ability of OX_1_ receptor signaling to produce 2-arachidonoyl glycerol, a CB_1_ receptor ligand, and a subsequent co-signaling of the receptors (Haj-Dahmane and Shen, 2005; Turunen et al., 2012; Jäntti et al., 2013). However, this does not preclude dimerization.

Phosphorylation (or other similar protein interaction) differences may be seen between the variants. Hydroxyl group-containing amino acids Ser, Thr and Tyr may be direct substrates for phosphorylation, but other amino acids can also affect the kinase consensus sequences. These sites have not been targeted in the point mutagenesis studies. Scansite (http://scansite.mit.edu/) (Obenauer et al., 2003) motif search suggest that some of the polymorphisms at OX_1_^167^, OX_1_^265^, OX_1_^279^, OX_1_^280^, OX_1_^408^, and OX_2_^401^ (Figures 1, 2) may impact kinase or other protein binding. Because these predictions are solely based on the amino acid sequence, however, they should be treated with caution. It is also unclear whether all these sites, especially OX_1_^167^, are accessible for interaction.

### Impact of genetic variation on the orexin peptide and receptor processing, folding and half-life

It is very difficult to predict the sites affecting receptor folding; in principle, every residue can influence receptor folding on the local or a more general level. A major change in the amino acid size, conformation, polarity and, especially, charge is likely to have a more pronounced effect of this type. Such an impact could be most pronounced for OX_1_^279^ Arg/Glu. Glycosylation, found on the extracellular GPCR surfaces, could be affected by the availability of Asn and Ser/Thr residues (and other sites in the putative consensus sequence). This could be relevant for the OX_2_^10^ Pro/Ser, OX_2_^11^ Pro/Thr, and OX_2_^193^ Cys/Ser variants (Figures 1, 2).

Posttranslational modifications are also necessary for the peptides for proper ligand function. For orexins, these include cyclization of N-terminal Glu and correct formation of the two disulphide bridges in orexin-A, and amidation of the free C-terminus of both peptides (Sakurai et al., 1998). With the very limited knowledge of the processing of orexin peptides and receptors as well as the very few studies which evaluate the orexin receptor variants in heterologous expression systems, it is difficult to predict a role for most of these processes in human disease. The one notable exception is the Leu16Arg mutant of the signal peptide of PPO, which impairs the processing of the PPO (Peyron et al., 2000), stressing the importance of the signal sequence for correct translation, packing and proteolytic processing.

For both receptors and peptides, the amino acid sequence may impact the trafficking and half-life. Receptor internalization from the plasma membrane is involved both in signaling and degradation, and requires interaction with other proteins (see above). Therefore, mutations may, for instance, decrease or increase the half-life of the receptor protein or redirect signal cascades.

## Orexin pharmacogenetics

Coding and non-coding variants of the OX_1_ and OX_2_ orexin receptors have been identified in many human populations and phenotypes. Due to the emerging market for drugs targeting OX_1_ and OX_2_ receptors, knowledge of genetic variation in the human genes coding for these receptors, *HCRTR1* and *HCRTR2*, respectively, is of pharmacogenetic interest. Although canine *HCRTR2* mutations are associated with narcolepsy (Lin et al., 1999; Hungs et al., 2001), mutations in human orexin receptor genes have been associated only with rather moderately elevated disease risks—and, in some cases, the associations have not been met with consensus. Table 1 presents a selection of the studies of OX_1_ and OX_2_ orexin receptor variants examined in human disease states.

**Table 1:** **Summary of OX_1_ and OX_2_ orexin receptor variants: investigations in disease**.

**OX_1_ aa**	**Corresponding aa in OX_2_**	**Location**	**SNP (or other access code)**	**Numbering/peyron**	**Numbering/olafsdottir**	**Findings**	**References**
167 Gly/Ser	Ile	TM4	rs144603792		652 G/A	Not linked with narcolepsy	Olafsdottir et al., 2001
265 Leu/Met	^a^	IC3		793 C/A		Not linked with narcolepsy	Peyron et al., 2000
279 Arg/Gln	^a^	IC3	rs7516785		989 G/A	Not linked with narcolepsy	Olafsdottir et al., 2001
280 Gly/Ala	^a^	IC3	NP_001516 (C), AF041243 (G)				
281 Arg/His	^a^	IC3	rs41439244	842 G/A	995 G/A	Not linked with narcolepsy	Peyron et al., 2000; Olafsdottir et al., 2001
408 Ile/Val	^a^	C-terminus	rs2271933	1222 G/A	1375 G/A	Not linked with narcolepsy	Peyron et al., 2000; Olafsdottir et al., 2001
						A allele associated with 1.4-fold risk of migraine	Rainero et al., 2011b
						1.6-fold increased risk of major mood disorders	Rainero et al., 2011a
						Association with polydipsia–hyponatremia in schizophrenia possible	Meerabux et al., 2005; Fukunaka et al., 2007
						No allele associations in panic disorder	Annerbrink et al., 2011
**OX_2_ aa**	**Corresponding aa in OX_1_**	**Location**	**SNP**	**Numbering/peyron**	**Numbering/olafsdottir**	**Findings**	**References**
10 Pro/Ser	Pro	N-terminus	rs41271310	28 C/T	352 C/T	Not linked with narcolepsy	Peyron et al., 2000; Olafsdottir et al., 2001; Thompson et al., 2004
11 Pro/Thr	^a^	N-terminus	rs41271312	31 C/A	355 C/A	Not linked with narcolepsy	Peyron et al., 2000; Olafsdottir et al., 2001
						Tourette syndrome/ADHD/sleep disorder 1.6 and 2.7-fold shifts in EC_50_ for orexin-A and -B	Thompson et al., 2004
193 Cys/Ser	^a^	TM4		577 T/A		Not linked with narcolepsy	Peyron et al., 2000
293 Ile/Val	^a^	IC3			1201 G/A	Not linked with narcolepsy	Olafsdottir et al., 2001
308 Ile/Val	Val	TM6	rs265334	922 G/A	1246 G/A	Not linked with narcolepsy	Peyron et al., 2000; Olafsdottir et al., 2001
						Ile variant associated with panic disorder in female patients	Annerbrink et al., 2011
						Migraine not associated. Association with CH found in some but not in all studies	Baumber et al., 2006; Schürks et al., 2006; Rainero et al., 2008
						Does not affect migraine drug response	Schürks et al., 2007a
401Thr/Ile	^a^	C-terminus		1202 C/T		Not linked with narcolepsy	Peyron et al., 2000

*aa, amino acid; IC, intracellular loop; SNP, single nucleotide polymorphism; TM, transmebrane helix*.

a *The corresponding amino acids can be defined with certainty only for TM regions*.

**Figure 3:**
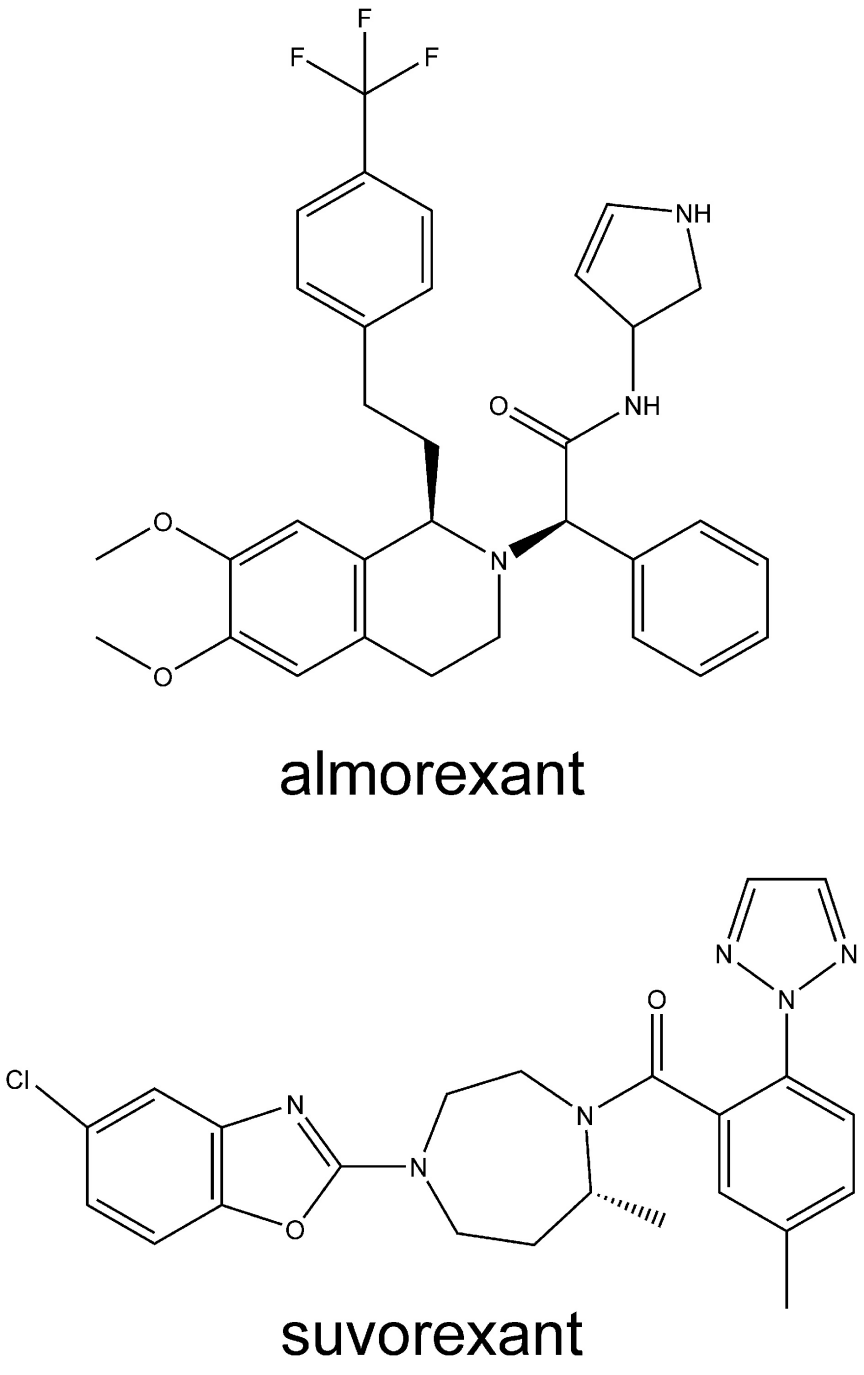
**Structures of some orexin receptor antagonists developed for insomnia**. Structures are rendered using ChemBioDraw Ultra 13.0 (PerkinElmer, Waltham, MA, USA).

With respect to drug development, the orexin receptor variants may be particularly relevant. Dual orexin antagonists, such as almorexant and suvorexant (Figure 3), as well as OX_2_-selective antagonists, have been developed for use as sleep aids. Orexin receptor antagonists seem to act to turn off wakefulness instead of inducing sleep, *per se* (Winrow et al., 2011). While the development of almorexant by Actelion Pharmaceuticals has been curtailed, Merck and Co. has continued the development of suvorexant (MK-4305) (Cox et al., 2010; Mieda and Sakurai, 2013; Winrow and Renger, 2014). Suvorexant completed three Phase III trials in 2013 (Winrow and Renger, 2014). The U.S. Food and Drug Administration's (FDA) peripheral and CNS advisory committee found the drug generally safe and effective for treating sleep maintenance and latency. Although it has a promising side-effect profile, the FDA review suggested that suvorexant is associated with increased somnolence the day after use, and that higher doses may be associated with an increased rate of suicidal ideation (Mieda and Sakurai, 2013). Pharmacogenetic considerations may assist in establishing the correct dosing for patients. Conversely, orexin receptor-activating therapy may become available for narcolepsy. Some recent findings support this therapeutic concept (Liu et al., 2011; Kantor et al., 2013). Narcolepsy may, however, not be the only disorder where such therapy may be beneficial, as indicated by some results briefly presented below and in Kukkonen (2012) and Kukkonen (2013).

Given the frequency of OX_1_ and OX_2_ receptor amino acid sequence variants, there is a clear rationale for examining the pharmacology of the agents at the variants *in vitro*. These results may, in turn, justify studying drug response in patients carrying receptor variants. For many human GPCRs, amino acid variants are well known to confer distinct pharmacological properties (Thompson et al., 2005, 2008a); however, the pharmacological data is generally unavailable for the human orexin receptor variants (Kukkonen, 2013).

## Orexin receptor variants identified in sleep/wakefulness disorders

Genetic variants of the orexin system have been identified only rarely in narcolepsy. For example, the Leu16Arg mutation in the signal peptide of PPO (Peyron et al., 2000; Gencik et al., 2001) disrupts a region of neutral, hydrophobic polyleucine amino acids in the PPO, which, in turn, limits the biologically active products, orexin-A and -B (Peyron et al., 2000). In contrast, the DNA or amino acid sequence variants of human HCRTR1/OX_1_ and HCRTR2/OX_2_ receptors (Figures 1, 2) do not seem to be involved in narcolepsy (Peyron et al., 2000; Olafsdottir et al., 2001)

OX_2_^11^ Thr variant was identified in two DQB1^*^0602-negative excessive daytime sleepiness (EDS) patients and an OX_2_^10^ Ser variant in a Tourette's syndrome patient comorbid with attention deficit hyperactivity disorder (ADHD) (Thompson et al., 2004). OX_2_^10^ Ser or OX_2_^11^ Thr variants were not identified in the 110 control individuals assessed, suggesting a possible association with sleep disorders. The fact that Tourette's syndrome patients diagnosed comorbid with ADHD frequently experience sleep disorders (Allen et al., 1992; Freeman et al., 2000; Cohrs et al., 2001), suggest that the OX_2_^10^ Ser might be involved in the aetiology of some sleep abnormalities. Furthermore, while the OX_2_^10^ Ser and OX_2_^11^ Thr variants were reported to be more common in HLA DQB1^*^0602-negative narcoleptics compared with controls, these variants were reported to be benign with respect to narcolepsy (Peyron et al., 2000). The presence of these variants in EDS and Tourette's syndrome patients, however, suggests that they should be evaluated *in vitro* for functional differences that may contribute to sleep dysregulation. In this context, it is interesting to note that orexin-A/orexin-B pharmacology was suggested to be altered in OX_2_^10^ Ser as compared to the wild-type OX_2_^10^ Pro variant (see Impact of the genetic variation on the orexin receptor ligand binding).

Thus, orexin receptor sequence variation may contribute to sleep disorders (OX_2_^11^ Thr, possibly OX_2_^10^ Ser). This is by no means surprising; it may rather be unexpected that so few findings of this type have been described. However, dramatic orexin receptor mutations might have such a detrimental effect on the regulation of wakefulness that they have been eliminated from the population, while milder phenotypes persist.

## The role of the orexin system in headaches

The orexin system has been examined in various forms of headache including migraine and cluster headache. While cluster headache is a rare, extremely debilitating headache that occurs in groups (or clusters) during seasonal changes (Goadsby, 2002), migraines are comparatively common headaches that are comparatively more treatable with non-steroid anti-inflammatory drugs or specific serotonergic drugs. Migraines often run in families where as cluster headaches are more sporadic (Benoliel and Eliav, 2013). While genome-wide studies have resulted in better understanding of migraine (Thompson et al., 2012), many complex traits, including cluster headache, have been less amenable to study—possibly due to their heterogeneity (Rainero et al., 2011b).

Cluster headache consists of attacks of sudden, severe, unilateral periorbital pain, accompanied by restlessness and cranial autonomic symptoms, and is characterized by a strikingly unique circadian and circannual rhythmicity (Goadsby, 2002). There is increasing evidence that cluster headache may have a significant genetic component. Genetic epidemiological studies have suggested that first-degree relatives of cluster headache probands are more likely to have cluster headache than the general population (Russell et al., 1996; Leone et al., 2001; El Amrani et al., 2002). Cluster headache has been found to be an autosomal dominant trait in some affected families, but despite numerous attempts, no clear molecular genetic basis has been identified for cluster headache (Russell, 2004). Segregation analysis suggests that cluster headache is a complex disease: several genetic factors may result in cluster headache when the correct environmental conditions are present (Pinessi et al., 2005).

Since there is no major gene effect for the orexin *loci* linked to migraine, the orexins have tended to be studied more frequently in cluster headache, where a model of complex genetic traits suggests multifactorial involvement that may include orexin *loci*. Numerous common polymorphisms of *HCRTR1* and *HCRTR2* have been studied in cluster headache (below), though the frequency and physiological significance of these variants is still being investigated (Rainero et al., 2004; Schürks et al., 2007a; Rainero et al., 2011b).

A number of studies provide evidence that orexins are involved in pain modulation within brain structures in the midbrain, indicating a possible link between orexins and the nociceptive phenomena observed in primary headache disorders (Bingham et al., 2001; Kajiyama et al., 2005; Mobarakeh et al., 2005). Orexin-producing neurons of CNS are also specifically located to the postero- and dorsolateral hypothalamus, regions implicated in cluster headache (May et al., 1998, 1999). The role of orexins in noradrenergic activation, shown by innervation and stimulation of the locus coeruleus in rats, monkeys and cats (Hagan et al., 1999; Horvath et al., 1999; Diano et al., 2003), may implicate changes in blood flow in cluster headache, although this evidence is not conclusive (Cohen and Goadsby, 2004). This suggests complexity of the cluster headache etiology.

### OX_1_ receptors in migraine

In addition to synonymous SNPs (rs10914456, rs4949449), a non-synonymous *HCRTR1* polymorphism of G1222A in exon 7 (rs2271933) encoding an OX_1_ Ile408Val substitution, has been implicated in migraine. Genotype and allele frequencies of the rs2271933 non-synonymous polymorphism have been associated with a 1.4-fold risk of migraine (Rainero et al., 2011b). The functional significance of this common OX_1_ receptor variant is not fully understood, however. While OX_1_^408^ in the receptor's C-terminus resides is likely to be located within the region for interaction with other proteins (below), it has not been experimentally resolved whether the mutation changes the receptor's expression level, coupling to effectors, or homo- or heterodimerization. Neither OX_1_^408^ variant correlates with human narcolepsy (Peyron et al., 2000; Olafsdottir et al., 2001).

### OX_2_ receptors in cluster headache

While the literature on the OX_2_/*HCRTR2* polymorphisms (rs1049880, rs3122156T, rs9357855G, rs2653342A, rs2653349G) is large, it remains inconclusive (Rainero et al., 2004, 2008). In particular, homozygosity for the common G allele of rs2653349 (1246 G/A), encoding OX_2_^308^ Val, has been associated with an increased disease risk for cluster headache—as compared to the OX_2_^308^ Ile variant—in some, but not all studies. The association was confirmed in a large study from Germany—showing that homozygous carriers of the G allele had a 2-fold increase in cluster headache risk (Schürks et al., 2006)—but not in a GWAS study of cluster headache patients of Danish, Swedish, and British origin (Baumber et al., 2006).

Additionally five intronic polymorphisms, covering more than 75% of the entire 108.35 kb sequence of the *HCRTR2* gene, were used to evaluate the association between cluster headache and the OX_2_/*HCRTR2* (Rainero et al., 2008). A significant difference between cluster headache cases and controls was found for 3 out of the 5 examined polymorphisms. Carriage of the GTAAGG haplotype—defined as a combination of the intronic positions Rs2653349, Rs3122156, Rs9357855, Rs2653342 and Rs3800539, and the exonic Rs2653349 (above)—was shown to be associated with cluster headache, resulting in a 3.7-fold increased risk. Sequence analysis of genomic DNA for the entire coding region of the OX_2_/*HCRTR2* gene in 11 cluster headache patients, identified no additional coding sequence difference besides rs2653349. The functional relevance of these intronic variants and how they impact cluster headache, remains to be determined (Rainero et al., 2008). In another study, neither one of the rs2653349 alleles (OX_2_^308^ Val or Ile) was associated with response to drugs, such as triptans, in cluster headache (Schürks et al., 2007a).

In conclusion, orexin receptor variants OX_1_^408^ Val and OX_2_^308^ Val have been associated with a somewhat elevated risk for migraine and cluster headache, respectively. While the intracellular OX_1_^408^ may affect the receptor interaction with other intracellular or plasma membrane proteins, and the TM6 OX_2_^308^ ligand binding, it is not known whether variants at these amino acids result in distinct functional properties, or if the difference might be found in differential processing etc., as suggested by co-segregation of OX_2_^308^ Val with the intronic *HCRTR2* polymorphisms. Future studies should address the significance of co-inheritance of variants of both receptors.

## Orexin receptor variants in psychiatric disorders

### Anxiety disorders

The role of the orexins in anxiety disorders is currently unclear because orexins have been suggested to be both anxiogenic and anxiolytic—or possibly even neutral—in investigations in rodent models (Kukkonen, 2013). Nevertheless, orexinergic interventions into panic disorder are currently under investigation (Perna et al., 2011). Panic disorder is an anxiety disorder characterized by unexpected attacks of fear with enhanced arousal and somatic symptoms (Meuret et al., 2011). The heritability of panic disorder has been estimated to be 50% (Hettema et al., 2001); however, the inheritance is most likely multifactorial. The rational for examining the involvement of orexins in panic disorder also reflects indications of orexin-mediated regulation of respiration (Kuwaki, 2008; Williams and Burdakov, 2008)—dysregulation of respiration is a hallmark of panic attacks. Orexin neurons innervate brain nuclei, such as the reticular formation of the medulla, that control respiration (Peyron et al., 1998). PPO knockout mice exhibit a decreased response to CO_2_ that is partly restored by supplementation with orexin (Deng et al., 2007). OX_1_ receptor antagonist, SB-334867, mimics the PPO knockout phenotype when administered to wild-type mice. Intracerebroventricular administration of orexin promotes respiration in rodents (Zhang et al., 2005; Johnson et al., 2012); in rats, this has been shown to be blocked by SB-334867 (Johnson et al., 2012).

OX_2_^308^ Ile variant has been associated with panic disorder in female patients (Annerbrink et al., 2011). In contrast, neither male nor female populations showed a risk associated with either variant (Val or Ile) of OX_1_^408^ (Annerbrink et al., 2011). Functional analysis of variant orexin receptors may provide insight into these allele associations.

### Mood disorders

Studies in rodents have suggested that orexins may also be involved in the pathogenesis of mood disorders. Wistar-Kyoto rats, an animal model of depression, have a reduced number of hypothalamic cells expressing orexin immunoreactivity (Allard et al., 2007). Furthermore, neonatal administration of the tricyclic antidepressant clomipramine may result in decreased orexin concentrations in brain regions such as the hypothalamus (Feng et al., 2008).

Consistent with this, intracerebroventricular administration of orexin-A has a long-term antidepressessive effect in some rodent depression paradigms; an effect that may involve hippocampal neurogenesis (Ito et al., 2008). Activation of orexinergic neurons leads to the excitation of major monoaminergic nuclei of the brain stem, including raphe nuclei (serotonin), locus coeruleus (norepinephrine), and the ventral tegmental area (dopamine) (Peyron et al., 1998; Leonard and Kukkonen, 2014). Furthermore, orexin knockout mice show a significant reduction in the dopamine turnover rate and a compensatory increase of serotoninergic activity possibly suggesting a relationship between monoamines and orexins (Mori et al., 2010).

The role of orexins in drug addiction and reward (Aston-Jones et al., 2010; Boutrel et al., 2010; Thompson and Borgland, 2011) may be linked to the proposed involvement of orexins in mood regulation, and the tendency of depressed patients to self-medication. The few studies conducted in humans suggest decreases in CSF orexin-A concentrations or its rhythmicity in depression (Salomon et al., 2003; Brundin et al., 2007).

The possible contribution of orexin receptor variants to axis I disorders, however, has not been widely studied. For example, the functional OX_2_^11^ Ser variant identified in Tourette's syndrome has not been examined in other patient cohorts. By comparison, studies of the OX_1_^408^ Val variant have reported it to be associated with a 1.6-fold increased risk of major mood disorders as compared to the OX_1_^408^ Ile (Rainero et al., 2011a). Patients homozygous for the Val-coding allele (1222A; rs2271933)—in comparison with those homozygous for the Ile-coding allele (1222G)—have an even higher, 2.5-fold increased risk for mood disorders, as also confirmed by haplotype analysis (Rainero et al., 2011a). Functional analysis of variant orexin receptors may confirm the relevance of these findings.

### Schizophrenia

OX_1_^408^ Ile/Val polymorphism has been studied in schizophrenia patients. Specifically, variation was associated with polydipsia–hyponatremia in schizophrenic patients when compared with non-polydipsic patients (Meerabux et al., 2005; Fukunaka et al., 2007); however, the associations were found for the opposite variants in the two studies.

Orexin regulation of water intake has been suggested by the anatomy of orexinergic projections to, for instance, subfornical organ and area postrema (Kukkonen et al., 2002). Intracerebroventricular infusion of orexin-A, more potently, but also of orexin-B, acutely increases water intake and drinking in rats (Kunii et al., 1999; Rodgers et al., 2000; Takano et al., 2004; Zheng et al., 2005; Kis et al., 2012), while in the continuous treatment (7 or 14 days), the effect wanes (Lin et al., 2002). PPO mRNA expression in the hypothalamus is upregulated upon water deprivation (Kunii et al., 1999). Orexin-A also excites subfornical organ neurons (Ono et al., 2008). Orexin-A alone does not affect baseline vasopressin release, but inhibits histamine-induced release in OX_1_ receptor-dependent manner (Russell et al., 2001; Kis et al., 2012). In golden hamster, infusion of orexin-B, but not orexin-A, in the amygdala stimulates water intake (Avolio et al., 2012).

Thus, one physiological role of orexins may be in the regulation of water intake. Whether this has a role in schizophrenia-associated polydipsia, remains to be shown. However, the dopamine D_2_-type receptor agonist, quinpirole, increases water intake, which is further enhanced by blocking OX_1_ receptors with SB-334867 (Milella et al., 2010), which would rather suggest an opposite role for orexin signaling under these conditions.

## Conclusions and perspectives

Human orexin receptor sequence variation has been investigated in the context of disease-based targeted approaches, first with narcolepsy and then with other diseases. Variants have been identified, some with an apparent relevance for the disease(s) under investigation, some not. Interestingly, the same variants that are putatively associated with headaches, namely OX_1_^408^ and OX_2_^308^ have been implicated in panic disorder, mood disorders and in polydipsia in schizophrenia. The “overrepresentation” of these variants in many disorders, however, may be an artifact from the candidate gene rational used to select these *loci*. However, in none of the cases has a very high risk been identified. Nevertheless, we suggest that the full functional phenotype of each receptor variant should be established; currently of greatest interest are the OX_1_^408^, OX_2_^10^, OX_2_^11^, and OX_2_^308^ variants. In the absence of these data, the reasons behind the observations are difficult to speculate on.

So far, none of the orexin receptor variants has conferred a very high disease risk or apparently a very grave phenotype. Is this because the two orexin receptor subtypes are simply too redundant; are the data sets confounded by a lack of valid disease phenotype classifications and an inadequate consideration of contributing environmental factors; or are the phenotypes carried by the known human orexin receptor gene variants simply rather mild? Currently, we cannot resolve this dilemma. We might assume, however, that any genotype grossly hampering orexin receptor function would be eliminated from the population due to its strong impact on the wakefulness, and thus the genotypes present only confer mild phenotypes. In essentially all cases we have to recognize the fact that cellular phenotypes of the orexin receptor variants have not been investigated, and conclusions based on this are thus vague. Evidence for a significant association of certain haplotypes of orexin genes with disease will inevitably be re-evaluated from a genome-wide perspective (GWAS) and with respect to the risk factors posed by other genetic and environmental variables. Although orexin receptor mutations may, alone, not confer highly increased risk (or protection) for a disease, these findings may propel studies concerning the physiological roles of orexins, and therefor also identify novel therapeutic approaches.

More complete study of orexins pharmacogenetics may facilitate novel areas of orexin research. This approach may help to refine drug design by targeting variant receptors. The continued study of orexin pharmacogenetics and receptor function at the cellular level is necessary before the role of orexinergic ligands in the treatment of disorders such as migraine, cluster headache, EDS and even idiopathic narcolepsy can be predicted fully. The emergence of dual orexin receptor antagonists, as well the possible OX_2_-selective ones (Dugovic et al., 2009; Etori et al., 2014), reinforces evidence for the partly overlapping and partly distinct roles of orexin receptors in the regulation of sleep/wakefulness states. Characterization of variant receptor pharmacology may be of further use in establishing pharmacogenetic profiles for the drugs. Specific groups of sleep disorder patients may benefit from these compounds. For example, the sleep disturbances characteristic of Parkinson's and Alzheimer's diseases may be amenable to orexinergic drugs. Parkinson's disease patients frequently complain of sleep disturbances and loss of muscle tone during rapid-eye-movement (REM) sleep. A more complete evaluation of the pharmacology of orexin receptor variants may facilitate the development of orexin receptor agonists.

## Author contributions

Every author contributed to the writing. Jyrki P. Kukkonen made the figures.

### Conflict of interest statement

The authors declare that the research was conducted in the absence of any commercial or financial relationships that could be construed as a potential conflict of interest.
